# Controversies in CPT® coding in the neonatal intensive care unit: - critical vs. intensive care

**DOI:** 10.1038/s41372-023-01704-6

**Published:** 2023-06-24

**Authors:** Satyan Lakshminrusimha, Clara Song, Stephen A. Pearlman, Gilbert Martin, Scott Duncan

**Affiliations:** 1 Department of Pediatrics, UC Davis Children’s Hospital, Sacramento, CA USA; 2 Southern California Permanente Medical Group, Pasadena, CA USA; 3 Department of Pediatrics, Sidney Kimmel Medical College at TJU, Philadelphia, PA USA; 4 Department of Pediatrics, Loma Linda University Children’s Hospital, Riverside, CA USA; 5 Department of Pediatrics, University of Louisville School of Medicine, Louisville, KY USA

**Keywords:** Paediatrics, Health occupations

## Abstract

Professional reimbursement to neonatal providers is based on the level of Current Procedural Terminology (CPT®) coding in the NICU, newborn nursery and other areas where neonatal care is provided. Four levels of evaluation and management (E&M) care—critical, intensive, routine-hospital care or normal newborn care can be provided to neonates. The work relative value units (wRVUs) associated with these four levels of care vary widely. This manuscript provides a brief review of basic features associated with each of these four levels with a specific perspective on differences between critical and intensive care codes. Coding and billing are constantly evolving fields with significant variation in interpretation and readers are encouraged to review the current publications on CPT® coding and make an informed decision on the best codes to be used for their patients.

## Introduction

The business of neonatology starts with a foundational knowledge of coding. Every neonatology group must oversee the coding process and may consider designating an individual to develop expertise and help supervise coding and billing. A healthy rapport with the business office engaged in coding and billing assures the utilization of correct codes which is important to sustain the financial viability of our practices. A recent article summarized a national survey and demonstrated the relative lack of knowledge that is necessary for accurate coding in neonatal practice [1]. Self-coding and having taken a course or webinar about coding were associated with higher coding accuracy.

The Current Procedural Terminology (CPT®) system [2], developed by the American Medical Association (AMA) in 1966, is revised annually and uses descriptive terminology to communicate work performed by clinicians to insurers [3, 4]. Each CPT® code is assigned a work relative value unit (wRVU, Table 1) [2]. Neonatal coding [5, 6] predominantly involves global daily codes, which are bundled to include the most common procedures and can be used once per 24-hour period. These codes are based on the age of the patient and level of service [2, 3]. The definitions of the specific codes are often a topic of conversation. In particular, the selections for critical versus intensive care service codes may be of greater importance to the practicing neonatologist as well as the business office.

**Table 1:** Common daily global codes in newborn care [2, 3].

CPT code	Description	wRVU
Critical care		
99468	Initial inpatient critical care, per day for the E&M of a critically ill neonate, 28 days of age or younger	18.46
99469	Subsequent inpatient critical care, per day for the E&M of a critically ill neonate, 28 days of age or younger	7.99
99471	Initial inpatient critical care, per day for the E&M of a critically ill infant or young child, 29 days through 24 months of age	15.98
99472	Subsequent inpatient critical care, per day for the E&M of a critically ill infant or young child, 29 days through 24 months of age	7.99
Intensive care		
99477	Initial hospital care, per day for the E&M of the neonates 28 days of age or younger, who requires intensive observation, frequent interventions, and other intensive services.	7.00
99478	Subsequent intensive care, per day for the E&M of the recovering VLBW infant (present body weight < 1500 g)	2.75
99479	Subsequent intensive care, per day for the E&M of the recovering LBW infant (present body weight 1500–2500 g)	2.50
99480	Subsequent intensive care, per day for the E&M of the recovering infant (present body weight 2501–5000 g)	2.40
Hospital care		
99221	Initial hospital care, per day, for the E&M of a patient, which requires medically appropriate history and/or examination and medical decision making (MDM) of straightforward or low (99221), moderate (99222) or high (99223) complexity. (These can also be time based – 99221 ≥ 40 min, 99222 ≥ 55 min, 99223 ≥ 75 min)	1.63
99222		2.60
99223		3.50
99231	Subsequent hospital care, per day for the E&M of a patient, which requires medically appropriate history and/or exam (straightforward or low complexity MDM–99231, moderate complexity MDM 99232 and high complexity MDM -99233) (These can also be time based 99231 ≥ 25 min, 99232 ≥ 35 min, 99233 ≥ 50 min)	1.00
99232		1.59
99233		2.40
Normal newborn care		
99460	Initial hospital or birthing center care, per day, for evaluation and management (E&M) of normal newborn infant.	1.92
99462	Subsequent hospital care, per day, for E&M of normal newborn	0.84
99463	Initial hospital or birthing center, per day, for E&M of normal newborn infant admitted and discharged the same day.	2.13
Discharge		
99238	Hospital discharge day management; 30 min or less	1.50
99239	Hospital discharge day management; more than 30 min	2.15

REF: https://www.cms.gov/medicaremedicare-fee-service-paymentphysicianfeeschedpfs-relative-value-files/rvu23a

The International Classification of Diseases (ICD) is designed to provide compatibility in the collection, processing, classification, and presentation of mortality and morbidity statistics across the globe [7]. The ICD has been revised periodically to incorporate changes in the medical field and the tenth revision (ICD-10) is currently in use in the US since October 1, 2015. Although the eleventh revision (ICD-11) was published by WHO in 2019, it has not been adopted in the US. Changes from ICD-10 to ICD-11 include new diagnostic codes, refinement of diagnostic criteria for existing diagnosis and steps in the direction of dimensionality (increase number of pertinent features) for some diagnoses. The ICD-10 provides various sections to include detailed codes for various diseases. For example, conditions originating in the perinatal period have codes ranging from P00-P96. Using the most specific code for the patient’s condition is critical for proper reimbursement. Instead of using a common code for respiratory distress (P22), being more specific and using P22.0 for respiratory distress syndrome (RDS) of newborn or P22.1 for transient tachypnea of newborn (TTN) will enhance diagnostic accuracy and assure proper reimbursement. ICD-10 codes are diagnostic codes that must support the CPT® codes being submitted for reimbursement. If there is a disconnect between the diagnosis and the billing code, insurance carriers will issue a denial.

## Global, bundled daily codes in neonatal care

The global, bundled daily codes used in neonatal care are listed in Table 1. In the order of decreasing complexity, the following global codes are commonly used in neonatal care.

Critical care: [2, 3]. A critical illness or injury is defined by CPT® as one that “acutely impairs one or more vital organs such that there is a high probability of imminent or life-threatening deterioration of the patient’s condition”. Critical care is the “direct delivery by a physician(s) or other qualified health care professional of medical care for a critically ill or critically injured patient”. Critical care codes are utilized when the case meets both of the following criteria: (a) Critical illness or injury as defined by CPT® listed above; and (b) the treatment delivered involves “high-complexity decision making to assess, manipulate, and support vital organ system function(s) to treat single or multiple organ system failure and/or prevent further life-threatening deterioration of patient’s condition. Coding critical care is not determined by the location of care, but services must be provided by a physician (of any specialty) or other qualified health professional (QHP). Many procedures such as umbilical vein or arterial catheterization, intubation, and surfactant administration are bundled in daily critical care codes. The procedures that are not included or bundled with daily critical care codes and thus may be reported separately are shown in Table 2.

**Table 2:** Common procedural codes that are not bundled with daily global codes in newborn care [2, 3].

CPT® code	Description	wRVU
33946	Extracorporeal membrane oxygenation (ECMO) or extracorporeal life support (ECLS) – Venovenous (VV) initiation	6.00
33947	ECMO/ECLS – Venoarterial (VA) initiation	6.63
33948	Daily management VV ECMO/ECLS	4.73
33949	Daily management VA ECMO/ECLS	4.60
99184	initiation of selective head or total body hypothermia	4.50
36568	Peripherally inserted central venous catheter (PICC) line placement	2.11
36680	intraosseous (IO) needle placement	1.20
92960	Cardioversion	2.00
92950	Cardiopulmonary resuscitation	4.00
38220	Bone marrow aspiration	1.20
36456	Partial exchange transfusion	2.00
36450	Double-volume exchange transfusion	3.50
32554	Thoracocentesis without imaging guidance	1.82
32555	Thoracocentesis with imaging guidance	2.27
32556	Chest tube without imaging guidance	2.50
32557	Chest tube with imaging guidance	3.12
49082	Abdominal paracentesis without imaging guidance	1.24
49083	Abdominal paracentesis with imaging guidance	2.00

REF: https://www.cms.gov/medicaremedicare-fee-service-paymentphysicianfeeschedpfs-relative-value-files/rvu23a

The following examples of conditions and interventions will typically qualify for critical care:

- Respiratory failure that requires invasive/noninvasive ventilation or nasal continuous positive airway pressure (CPAP).
- Hypotension, shock, or cardiac failure that requires inotrope and/or vasopressor therapy.
- Congenital heart disease needing intravenous prostaglandin E1 infusion.
- RDS requiring surfactant (either following intubation or less-invasive surfactant administration). Note: by ICD-10 definition, respiratory failure is inherent to the diagnosis of RDS.
- Hypoxemic respiratory failure or pulmonary hypertension or right ventricular cardiac failure that requires inhaled nitric oxide (iNO) therapy.
- Severe hyperbilirubinemia treated with double volume exchange transfusion.
- Symptomatic polycythemia requiring partial exchange transfusion.
- Acute tension pneumothorax or pneumopericardium or pleural or pericardial effusion resulting in acute life-threatening deterioration needing thoracentesis or thoracostomy tube/chest tube, or pericardiocentesis or pericardial drain placement (Note – placement of a chest tube or pericardial drain as part of a surgical protocol by itself does not justify a critical care code).
- Severe bradycardia or cardiac arrest in the NICU needing cardiopulmonary resuscitation (which at least includes chest compressions).
- Renal failure or acute tubular necrosis requiring therapeutic intervention.
- Necrotizing enterocolitis (Bell’s stage II or higher) and infant is on orogastric suction and not feeding.
- Moderate to severe hypoxic-ischemic encephalopathy (HIE) with therapeutic hypothermia (selective head or whole-body cooling).
- Status epilepticus or intractable seizures receiving antiepileptic therapy.
- Congestive cardiac failure (unstable) requiring frequent adjustment of medications and respiratory support.
- Post-operative management following general anesthesia if the infant needs respiratory support and frequent adjustment of cardiovascular and pain medications (appropriate modifiers must be used by the neonatologist if the neonatal surgical care code includes postoperative care). Discussion with hospital coding experts to discuss optimal care codes and modifiers by the surgical and neonatal providers may be appropriate in such circumstances.

Optimal documentation to justify a critical care code includes the use of terms such as “failure”, “imminent” and “life-threatening deterioration” to describe the patient’s illness. Imminent and life-threatening do not have a clear definition approved by CPT®. Established definitions for organ system failure such as respiratory failure (oxygenation or ventilation i.e., PaO_2,_ SpO_2_ or PCO_2_ outside the normal range requiring active intervention) should be used.

Intensive care refers to neonates and infants who do not meet the definition of critically ill but continue to require intensive observation, frequent interventions, and other intensive care services. This includes cardiorespiratory monitoring, continuous and/or frequent vital sign monitoring, heat maintenance, enteral and/or parenteral nutrition adjustment, laboratory and oxygen monitoring, and constant observation by the healthcare team under direct supervision of the physician or other QHP. These codes are commonly used for recovering low birth weight infants (“growing preemies”) who continue to require higher level of care for temperature instability, apnea/bradycardia monitoring, low-flow nasal cannula support, parenteral nutrition and orogastric or nasogastric feeds. If such intensive monitoring, care or supervision is not required, subsequent hospital care (99231–99233) or normal newborn care (99462) should be reported.

While the guidelines above provide an overall differentiation between critical and intensive care, deciding on the most appropriate level of care can be controversial and subject to interpretation. Appropriate documentation for the level of coding is crucial to ensuring approval by insurance carriers.

### Documentation

It is important to appropriately document notes in the electronic medical record (EMR) to justify critical or intensive care. The purpose of documentation is two-fold, to communicate the patient’s medical condition and to document the work performed to care for the patient. In addition to the usual elements of documentation including the relevant review of the history and physical examination, the critical care codes require the inclusion of specific information. The note must document that the patient is critically ill with organ failure, describe all the care provided and that it involves high-complexity medical decision making (MDM) to assess and support organ failure or to prevent life-threatening deterioration. The documentation for the intensive care codes should include, in addition to the same basic elements, a description of the patient’s medical condition and that they require continuous or frequent monitoring of vital signs or adjustments in therapy and constant observation by the healthcare team. In both critical and intensive care patients, the clinician must document that all aspects of the care are being delivered under their supervision. Suggested documentation at the end of a clinical note for critical and intensive care is shown in Table 3.

**Table 3:** Suggested documentation to justify critical and intensive care.

CRITICAL: This patient is experiencing vital organ impairment requiring support and interventions as delineated in the above problem list. Medical management including frequent assessments of patient status, medical decision making, and intervention adjustments of high complexity is required to prevent life-threatening deterioration in the patient’s condition.
INTENSIVE/WEIGHT-BASED: This patient is under constant supervision by the health care team and is requiring intensive cardiac and respiratory monitoring, including frequent or continuous vital sign monitoring, maintenance of neutral thermal environment and/or nutritional management. Current status and treatment are delineated in the above problem list.

Hospital care of the ill newborn that do not require intensive care or critical care services as outlined above but require an increased level of physician care, nursing observation, or physiologic monitoring are typically reported with hospital inpatient or observation care codes (initial 99221–99223 and subsequent 99231–99233, Table 1). Note that these are daily codes, with the correct code chosen based on complexity of MDM and/or time.

A normal newborn may be defined as an infant, birth through first 28 days [2], who has undergone normal transition after birth (irrespective of the nature of delivery room intervention received) [3]. Codes for initial, subsequent and same-day admission and discharge codes are shown in Table 1.

## Hourly critical care

There are some instances where hourly critical care codes (99291—first 30–74 min –4.50 wRVUs, 99292—subsequent critical care for each additional 30 min—2.25 wRVUs) are used. Critical care time accrues when the provider is face-to-face with the patient, including time spent directly related to patient care, such as reviewing test results, discussion with family and medical staff and documenting services in the EMR. Some bedside procedures that are bundled within the global critical care codes are not bundled with the 99291–99292 code set. The time spent performing these separately billable procedures cannot be included in the hourly critical care time. Critical care hourly codes can be used instead of or in addition to global daily critical care codes (Table 1) only in specific instances included below.

- Concurrent critical care provided by a second physician from a different specialty providing a unique service (e.g., cardiologist, pulmonologist, nephrologist or pediatric intensivist); similarly, a neonate in the Pediatric Intensive care unit (PICU) or cardiac ICU may need a neonatologist to provide critical care such as managing a high frequency jet ventilator.
- Transfer of care such as from the NICU to the cardiac intensive care unit following cardiac surgery (the neonatologist in the NICU reports hourly critical care code and the receiving cardiac unit intensivist reports daily global critical care code).
- Note: In order to use these critical care hourly codes, the second specialist must provide cumulative (can be continuous or intermittent in a given day) critical care for at least 30 min on the floor or unit. Time provided by different physicians or QHP of the same specialty and medical group can be combined to count for these services.
- Critical care services provided in preparation for transfer of the critically ill neonate to a different neonatology group and hospital system.

## Clarifications regarding critical and intensive care

### On-site provider presence

Traditionally, daily global critical and intensive care involve direct patient care and could not be provided through telemedicine or telephone. However, the Centers for Medicare and Medicaid Services (CMS) has made an exception under the ongoing public health emergency. All neonatal and pediatric critical and intensive care codes can be applied to services provided by telemedicine (see the following website for more details; this exception currently expires on December 31, 2023—https://www.cms.gov/medicare/medicare-general-information/telehealth/telehealth-codes). We recommend that providers check CMS website for guidance beyond December 31, 2023.

### Overnight in-house physician presence

Constant 24 h/day in-house attendance by the supervising physician is not a requirement for daily, global, bundled critical and intensive care codes. However, the wRVU assigned to these daily critical and intensive care codes were established under the assumption that the provider is physically present in the unit and is directly providing or actively participating and supervising patient’s care during a significant portion of the reported service. Justification of critical or intensive care, medically appropriate examination or assessment and active participation should be documented in the EMR.

### Neonatal vs. pediatric critical care codes

There is a difference between wRVUs for initial neonatal vs. pediatric critical care codes (18.46 vs. 15.98, respectively) although subsequent critical care carries the same weight (7.99, Table 1). Neonatal codes are used until 28 postnatal days (day of birth is day 0) and pediatric codes begin on or after 29^th^ postnatal day. For example, an infant born on 1/1/23 (day #0) will use neonatal codes until 1/29/23 (day #28) and pediatric codes from 1/30/23 (day #29) [3].

Initial neonatal (99468) and pediatric (99471) codes are to be used only once during each hospital stay. However, recognize that these are not admission codes. For example, an infant admitted to the NICU for hypoglycemia and receiving intensive care on postnatal days 0–2 develops sepsis and is intubated with inotropic support on postnatal day#3, an initial critical care code (99468) can be used on this day of service.

Initial neonatal intensive care code (99477) is an age-based code and can only be used in a neonate 28 days or younger regardless of current weight. If an infant 29 postnatal days or older is admitted to the NICU for intensive care, the initial day code for the day of hospital admission should be 99223 (if complex MDM is involved). In contrast to 99477, the subsequent intensive care codes (99478-80) are weight based and can be used beyond the neonatal period for infants whose current weight is ≤ 5000 grams and require intensive care services.

Subsequent intensive care codes (99478-80) are based on current weight. If a patient’s weight changes in either direction and crosses the weight threshold for a particular code, the appropriate code for that daily weight should be used. For example, an infant with a birth weight of 1500 g is admitted on Monday and required intensive care (code—99477). The current weight on Tuesday is 1500 g and on Wednesday is 1499 g. The intensive care code for Tuesday will be 99479 but Wednesday will be 99478.

### Subsequent Intensive care >5000 g

There are no subsequent intensive care codes when the infant’s current weight exceeds 5000 g. It is recommended that an appropriate subsequent hospital care code be used for these infants. As shown in Table 1, the wRVUs for subsequent intensive care for 2501–5000 g infant (99480) and high complexity hospital care code (99233) have similar wRVUs (2.40) per day. Initial intensive care code (99477) and critical care codes (99468 and 99469) do not have a weight limit and can be used for neonates with a current weight > 5000 g.

Discharge codes (99238—30 min or less and 99239 - >30 min) are time-based codes and are the same irrespective of critical, intensive, hospital or normal newborn care rendered to the neonate during the hospital stay. No other daily, bundled code can be used on the day of discharge regardless of the time of discharge. These codes are daily codes but are based on time; time for discharge examination, discussion with parents or guardians, discharge planning and communication with follow-up primary care and specialists, instructions for relevant caregivers such as home nursing, preparation of prescriptions, referral forms and discharge records. This time need not be spent on a continuous basis but can be cumulative on that calendar day. Recording a specific time (45 min) is better than stating >30 min. The same codes are used for the day of death.

### Transfer of care

If a patient is transferred within the same facility to a different location and is cared by the same physician group and subspecialty, a single daily global care code is used. Similarly, if a patient is being transferred to a different facility but is cared by the same physician group, a single daily global care code is used (at one of the two facilities only). If a patient is either acutely transferred or electively back-transferred from one facility to another, and cared by a different physician / QHP group, the following guidelines can be considered.

- Acute transfer to a higher level of care: If the critically ill patient is being transferred to another facility and if the care is taken over by a physician or QHP from a different medical group, hourly critical care codes (99291–99292) are used by the transferring physician and a daily global critical care code (99468 or 99471) can be used by the accepting physician or QHP. If the patient is not receiving critical care and is transferred to a tertiary care facility e.g., a three-month-old former 24-week gestation infant on nasogastric feeds and nasal cannula oxygen with a post-hemorrhagic hydrocephalus is transferred from a community NICU to the Children’s Hospital for a ventriculo-peritoneal shunt placement. The transferring physician uses a hospital care code (99231–99233). The accepting physician at the Children’s Hospital intubated the infant for surgery and bills for critical care.
- Back-transfer to a lower level of care: If a patient receiving intensive care (e.g., the infant in the above vignette was extubated after shunt surgery and is on a nasal cannula 1 LPM oxygen but on nasogastric feeds) is being transferred back to the community hospital, the transferring physician at the Children’s Hospital uses a routine care code (99231–99233) and the receiving physician at the community hospital bills a daily global intensive care code (99478–99480) or hospital care code (99231–99233). If the baby in the above example is receiving critical care (e.g., still intubated on mechanical ventilation after surgery), the transferring physician uses hourly critical care codes and the accepting physician uses global critical care admit code.
- Note – some insurance companies may deny the use of a daily global code for the same day by two different physician groups and deny one of these charges.

To summarize, the principles of coding for back-transfer are as follows: The transferring provider does not use daily critical or intensive care codes (with one exception—see # 2 below). The receiving provider typically uses daily codes.

1. Critical care –When the critically ill neonate or pediatric patient improves and is transferred to a lower level of care to another individual in another group within the same facility, the transferring individual does not report a per day critical care service. Subsequent hospital inpatient or observation care (99231–99233) or time-based critical care service (99291–99292) is reported, as appropriate, based upon the condition of the neonate or child. The receiving individual reports subsequent daily critical care (99469 or 99472), subsequent intensive care (99478–99480) or subsequent hospital inpatient or observation care (99231–99233) services, as appropriate, based upon the condition of the neonate or child.
2. Intensive care or routine hospital care—When the neonate or infant improves after the initial day and no longer requires intensive care services and is transferred to a lower level of care, the transferring provider does not report a per day intensive care service. Subsequent hospital inpatient or observation care (99231–99233) or subsequent normal newborn care (99460, 99462) is reported, as appropriate, based upon the condition of the neonate or infant. The exception to this is transfer on initial day of Intensive Care. If the transfer to a lower level of care occurs on the same day as initial intensive care services were provided by the transferring individual, 99477 may be reported. The receiving provider bills a global intensive care code (99478–99480) or hospital care code as appropriate (99231–99233).
3. Discharge code (99238–99239) does not apply for transfers since the baby is not going home.

Car seat testing codes (94780-for the first 60 min and + 94,781 for additional full 30 min) can be used with hospital care codes (99231–99233), normal newborn codes (99460–99463) or discharge codes (99238–99239) but not with subsequent intensive care codes (99478–99480).

## Areas of controversy

### High flow nasal cannula (HFNC)

The use of heated, intensely humidified, high flow through a nasal cannula is common in the NICU. The optimal code (critical vs. intensive) depends on severity of the illness and intensity of service. If HFNC is being used in the NICU to treat a critical condition such as respiratory failure due to RDS, bronchopulmonary dysplasia (BPD) or frequent apneic spells, and in the opinion of the provider, withdrawal of the HFNC support would lead to imminent or life-threatening deterioration with significant morbidity or mortality, a critical care code can be justified. The documentation in the EMR should reflect the above thought process. If such a justification cannot be documented, it is prudent to utilize an intensive care or hospital care code.

Apnea and Bradycardia of prematurity which is unstable in the opinion of the provider (multiple spells / 24 h with some spells requiring moderate or vigorous stimulation or mask ventilation) may be considered critical care. Many of these infants require respiratory support. However, if such spells do not require active intervention by the physician or the QHP, intensive care codes may be considered.

### Extreme prematurity

Infants at extremely low gestational age (<28 weeks) are at serious risk of mortality and morbidity. However, prematurity alone cannot justify critical care codes. The infant must exhibit a critical illness and require frequent assessment and intervention with high-complexity MDM as documented in the EMR to enable the use of a daily, global critical care code.

### Hypoglycemia

Late preterm and term infants are often transferred from newborn nursery or mother-infant dyad rooms to NICU for management of hypoglycemia. If infants require continuous intravenous glucose supplementation with frequent monitoring and multiple adjustments of IV glucose rates or if a central venous line (e.g., umbilical venous line) was placed to accommodate high concentrations of glucose, intensive care codes may be used. Persistent hypoglycemia (often due to hyperinsulinism) in the setting of elevated glucose infusion rates requiring the addition of diazoxide or glucagon may justify critical care coding. If glucose levels are stable and do not require frequent monitoring or adjustment, hospital care codes may be more suitable.

## Conclusion

Every neonatologist should have basic knowledge of neonatal coding. Differentiating between critical care and intensive care codes is important as the wRVU assignment for these codes is vastly different (Table 1) and relatively subjective. Appropriate justification of the code includes proper documentation of direct involvement in patient’s care, the level of illness of the patient, and the services provided. In the future, reimbursement based on aligned funds-flow [8], value-based care, healthcare costs [9], outcomes and patient satisfaction [10] may bring additional challenges to physician compensation.

## Disclaimer

The description and interpretation of CPT® codes is subject to change and readers are encouraged to review the most recent guidelines and recommendations. This manuscript is based on 2023 guidelines. Each neonatal provider should interpret the CPT® codes based on infant’s illness and care provided and make an informed decision on the appropriate code for service. In some instances, individual variation among insurance payors’ interpretation of CPT® codes may occur and discussion with billers, coders and insurance personnel might be beneficial.

## Data Availability

All information included in this review article is from public sources and CPT books published by the American Medical Association and American Academy of Pediatrics.
